# The Æsthetics of Suicide

**Published:** 1861-10

**Authors:** 


### Art. III.—THE ÆSTHETICS OF SUICIDE.

In a previous number of this Journal* we endeavoured to show that the poetical literature of this country did not, as certain continental writers maintain, exercise a primary influence in the development of that æsthetical treatment of suicide which has been one of the most curious phases of the literary history of the past eighty years. We attempted also to trace the early history of the modern æstheticism of suicide. We propose now to examine the latest manifestation of this æstheticism as exhibited in recent French literature.

In order more clearly to indicate the relationship existing between the earliest and latest examples of the æsthetical treatment of suicide among the moderns, it will not, perhaps, be amiss, in the first place, briefly to recapitulate the history of its development.

Throughout the latter half of the last century, a remarkable increase of suicide in Europe occurred contemporaneously with a progressive decline in the power of religion and of the Church over men's minds, a gradual deterioration of morality, and the

* No. II. Art. 2. *The Classic Land of Suicide.*

rise and spread of a philosophical scepticism, which undermined all that had previously been looked upon as necessary for the stability of society. Towards the termination of the century, as Carlyle has forcibly said, "Whatever belonged to the finer nature of man had withered under the Harmattan breath of Doubt, or passed away in the conflagration of open Infidelity; and now, where the Tree of Life once bloomed and brought forth fruit of goodliest savour, there was only barrenness and desolation."* A profound disquiet, a necessary consequence of the loosening of those holier ties which bind men to life, had sprung up widely in the minds of thoughtful persons, and begotten a life-weariness or indifference of which suicide was the logical and too often practical sequence.

It was under such circumstances that Goethe's *Sorrows of Werther* appeared. This work, chiming in with and giving for the first time a definite expression to the prevalent feeling of the moment, seized in a remarkable manner upon the hearts of men. Werther is the type of a refined but ill-regulated, ill-balanced spirit, which, imbued with the literature and scepticism of the epoch, conceives the chief aim of existence to consist in sensuous enjoyment. He acknowledges no higher duty than the gratification of his own pleasures, no higher rule of conduct than such as most readily falls in with the pursuit of that duty. He looks with contempt upon the pursuits, hopes, and pleasures of every-day life, and seeks an ideal life which clashes at every turn with the real. Hence ever-recurring dissatisfaction and disgust both with himself and with all around him. But, "hemmed in as he is, he ever keeps in his heart the sweet feeling of freedom, and that this *dungeon* can be left when he likes."†

These are the salient points of Werther's character, and consistently with them he is represented as cherishing an adulterous love for the wife of a friend, justifying that love, and to crown all, advancing its want of gratification as a seal of martyrdom, and a sure passport to eternal bliss.

"Everything," Werther writes, "passes away, but a whole eternity could not extinguish the living flame which was yesterday kindled by your lips, and which now burns within me. She loves me! These arms have encircled her waist, these lips have trembled upon hers. She is mine! Yes, Charlotte, you are mine for ever!

"And what do you mean by saying Albert is your husband? He may be as for this world: in this world it is a sin to love you—to wish to tear you from his embrace. Yes, it is a crime, and I suffer the punishment—but I enjoy the full delight of my sin. I have inhaled a balm that has revived my soul. From this hour you are

---

* Miscellanies; Art. *Goëthe.*

† Letter, 22nd May.

mine; yes, Charlotte, you are mine! I go before you. I go to my Father, and to your Father. I will pour out my sorrows before Him, and he will give me comfort till you arrive. Then will I fly to meet you. I will claim you, and remain in your eternal embrace, in the presence of the Almighty.

"I do not dream; I do not rave. Drawing near to the grave [he is writing on the eve of suicide] my perceptions become clearer. We shall exist; we shall see each other again; we shall behold your mother; I shall behold her, and expose to her my inmost heart. Your mother—your image."*

That the *Sorrows of Werther* should have been received with avidity on all hands, and rapidly multiplied in every Christian language, is one of the most significant indications which could be given of the peculiar state of society at the period in which the book was written. Apart from the charm which belongs to the great and enduring literary merits of the work, and from the fact that it first gave expression to the vague and undefined disquiet (the "nameless Unrest," as Carlyle has it) which had settled down upon men's minds, there is another and equally, if not more, important element which contributed to the immediate and amazing popularity of Werther. While the widely-spread scepticism of the period had unseated all the most cherished and holier beliefs of humanity, it had substituted nothing capable of satisfying those deep-rooted feelings which underlie, and are the source of those beliefs. The moral attributes of humanity having been frittered away, the conceptions of vice and virtue became regulated by individual or general expedience, and as a consequence, the former assumed a quasi-virtuous, the latter a quasi-vicious aspect. But a degraded virtue was ill adapted to satisfy the irrepressible yearnings of the heart after something purer than a mere sensuous delight in existence; and the virtuous garb in which vice had been clad sat too awkwardly upon it altogether to hide its revolting nature. Hence neither the thoughtful nor the thoughtless, who had yielded to or trifled with the philosophical scepticism of the time, felt at ease with doctrines which ignored, but could not extinguish, the deep-set faith that good and evil, right and wrong, were something more than mere matters of expediency; which were all-important to the vicious, but which left the virtuous in the lurch; and which reduced man to the position of being solely the most intellectual brute in creation.

Under such circumstances Werther came as a New Gospel. It threw a veil of exquisite sentiment over the heartless and repulsive teachings of scepticism, and seemingly linked them to

* Letter, Dec. 20.

the holiest feelings of our nature. With a subtle ingenuity worthy of the refinement which Mephistopheles subsequently boasted the devil had undergone as civilization had advanced,* Werther stopped short of avowed atheism, and substituted an æsthetical deity for the God of Christianity, pressing ingeniously into service the familiar phraseology of the Christian religion, and prostituting it to the service of vice. Thus Werther supplied that element of feeling which had been wanting in the scepticism of the day, and by retaining the idea of Deity and the language of religious faith, it apparently reconciled the profession of virtue to the practice of vice—neatly dovetailing the one into the other.

To what extent Werther influenced the educated classes of the people is best shown by the effect which the book exercised upon men of letters. " Infusing itself into the core and whole spirit of Literature," writes Carlyle, " Werther gave birth to a race of Sentimentalists, who have raged and wailed in every part of the world ; till better light dawned on them, or, at worst, exhausted Nature laid herself to sleep, and it was discovered that lamenting was an unproductive labour." Of these Sentimentalists Byron was the chief—the strongest, the wildest, and the gloomiest.

The impetus given to suicide by the publication of Werther was great. " The eternal law has done nothing better than this," Seneca wrote, " that it has given us only one entrance into life, but a thousand ways of escaping out of it. Excellent is the condition of human life ; since nobody can be miserable but by his own fault. Does life please you ? live on. Does it not ? go from whence you came. No vast wound is necessary ; a mere puncture will secure your liberty. It is a bad thing, you say, to be under a necessity of living ; but there is no necessity in the case. Why not ? because many, short and easy, are the paths to deliver you from it. Thank the gods, nobody can be compelled to live ; we spurn at such a necessity. If the mind be sick, it is its own fault ; it may soon put an end to its misery. Do you see that

---

* " Refinement, too, that smoothens all
O'er which it in the world has pass'd,
Has been extended in its call,
And reached the devil, too, at last.
That northern phantom found no more can be,
Horns, tail, and claws, we now no longer see ;
As for the foot—I cannot spare it,
But were I openly to wear it,
It might do greater harm than good
To me among the multitude.
And so, like many a youth beside,
Who bravely to the age appears,
Yet something still contrives to hide,
I've worn false calves for many years !"—*Faust.*

precipice, that river, that well? you will find liberty or freedom from misery at the bottom. Look on that tree; liberty hangs on its branch; or do you ask, which is the road to liberty? your heart, your throat, and every vein in your body. Every one ought to make his life approved by others, his death to himself. That kind of death is best which pleases most. If a man can contrive to kill himself easily and without much pain, he ought so to do; but if prevented from this, he must use more ingenious and painful methods. There never can want contrivance to die, where there is inclination. It is most unjust to live by theft, but to steal an opportunity of dying is very becoming and beautiful."*

In this wise also Werther taught the becomingness and beauty of suicide, and a like lesson flows, directly or indirectly, from the writings of the Sentimentalists. But it is worthy of note, that the ancient Stoics justified suicide solely as a means of escape from the ills which might overtake the virtuous—those of a just and approved life; but the modern Epicurean seeks to justify the act chiefly as a deliverance from the miseries induced by vicious habits.†

In England the moral atmosphere has been in a great degree purged of the suicidal infection arising from the Werther school of literature, but such is not the case in several continental States. In France, for example, works of this school have been multiplied, and their influence has, if anything, gained ground

---

* Ep. lxx.

† "The suicide of old (when his death assigned him to any degree of credit, and not rather to infamy) despatched himself, because he had been unsuccessful in the field of battle, had lost a town or an army; the modern one does the same because he has lost a throw on the dice; the former staked his life on the event of a battle, the latter does the same on the turn up of a card. The ancient hero put an end to his mortal existence rather than yield his person to grace the triumph of conquest; the modern man of honour departs out of life to defraud his injured creditors. The man of ancient virtue fought in the cause of public liberty and would not breathe after that expired; the man of modern dissipation as strenuously engages on the side of licentiousness, and when no longer able to maintain its dissolute empire, rushes indignant from the scene of sensual existence. Women of old died by their own hands to preserve an untainted chastity of manners; while the men of modern spirit voluntarily hazard their lives in defence of female prostitution. A disinterested love of their country led men of old to destroy themselves rather than offend against the dignity and authority of wholsome institutions; a complete love of themselves inspires many of the present generation to injure the laws of their country in a thousand shapes, and then to fly from the execution of their sentence by the stroke of self-murder. The suicide of the ancients was frequently grounded on some principle of genuine honour, dignity and patriotism, while the self-murder of the moderns too of ten originates in mere pride and disappointment, in sensuality, extravagance, and consequent penury. In short, as the one frequently arose from some exertion of probity and justice, from some principle of honest worth, so the other is to be traced to the haunts of the gamblers, to the brothels of vice and profligacy; it owes its birth to the extinction of virtuous principles, its constant and alarming growth to the destruction of all pious and religious impressions."—*Moore on Suicide*, vol. i. p. 284.

from year to year; and at the present time *Wertherism* (so to speak) flourishes vigorously in that country. Scepticism clothed in a garb of religious phraseology, sensuality culminating in the apotheosis of prostitution and adultery, and suicide elevated to the dignity of a legitimate panacea for all the ills of life, constitute the staple of perhaps the most popular portion of the current romance and dramatic literature of France. To the demoralizing effects of this literature, French writers of note* justly ascribe an all-important influence in the production of suicide—tracing to this source in no small degree the preponderance of suicides among the young in Paris (the chief focus of Wertherism), as well as the progressive increase in the amount of suicide throughout France since the commencement of the century.†

If we would learn the character of the existing æsthetical literature of suicide, a typical example is to be found in Maxime du Camp's *Mémoires d'un Suicide.*‡ This work is cast in a somewhat similar literary mould to the *Sorrows of Werther*, and its hero is a new Werther, after the most novel and approved Gallic model. We first make his acquaintance, in a highly melodramatic fashion, in Egypt, on the route from Keneh to Kosseir. Our author is travelling homeward from the Upper Nile and he stumbles across his future hero, who has been vainly seeking distraction in the depths of occidental Asia, in the desert of Kosseir. Pass we over, however, those incidents and details which, although pleasantly spicing the prefatory chapter of the work, have no immediate bearing upon its subject matter. Let it suffice that the new Werther is named Jean Marc, and that he is depicted as being in the prime of life, and as having the aspect of one who was a victim to insurmountable ennui. To use his own phrase, as he sits gossiping freely with his new-made friend at their halting-place :—" He had seized hold of life on the wrong side, and the penalty bore him down perpetually." " Nonsense," is the rejoinder, " every evil has its remedy within itself, and as good men say, every one carries his cross: do you know a happy man ?" " Yes," he said ; " I have seen one." " Where ?" quoth

---

* See the works of Brierre de Boismont and Lisle.

† See *Journal of Psychological Medicine*, vol. xii. p. 209. The tendency to suicide throughout England is greatest between the 55th and 65th years of life ; throughout France, between the 40th and 50th ; in Paris the tendency is greatest from the 20th to the 40th years ; in London (for the three years 1858—60) from the 40th to the 60th. In 1827 the proportion of suicides to population throughout France was 1 in 20,660. From that year the number underwent a steady increase, year by year, with but few exceptions, and in 1852 the proportion had reached 1 in 9,340.

‡ Mémoires d'un Suicide, recueillis et publiés par Maxime du Camp. Paris, 1855.

the friend; "the case is rare, and I would willingly seek him to learn his secret." "In a village of Nadj," answered Jean Marc. "He was a Koord who had been taken prisoner by the Wahabis, in the war against the Pasha of Egypt; he is condemned to turn the wheel of a Sakyeh from morning until evening. At sunrise he betakes himself to his rude labours, which he performs conscientiously, dreading the cudgel of his master; when night comes he lays himself down on a mat and sleeps soundly until the following morning." "And you think that this wretch is happy?" "'If I was still so foolish as to believe in happiness, I would seek it in habit.' So says Chateaubriand, and he is our master in all things. This wretch, as you call him, is habituated, therefore he is happy." "If it is so, it would be well to take his place." "No," is the reply; "because I should take the place without having acquired the habit, and so I should fail in my object." "You love paradoxes," said the friend, laughing. "Less than you believe," answered Jean Marc, with a sad and serious expression.

Thus they gossip in the desert, and Jean Marc is represented now as indulging in fatalistic doctrines borrowed from the Mahometans, and anon as contending for a transcendental conception of freewill — smoothing over the flagrant contradiction of opinions by the sententious observation that "All this could be reconciled" (in what manner we shall see in the sequel).

And so the conversation by an easy transition reverts to the subject of suicide. Is voluntary death permitted or prohibited? To this question Jean Marc responds in the affirmative. Upon which his interlocutor exclaims, "Is it right to withdraw any force whatsoever from the course of things?" "Certainly," said Jean Marc, "if that course carries me where I do not wish." "That is fatalism." "Yes," is the rejoinder; "but I kill myself as an act of freewill, and thus I re-establish the equilibrium." And further Jean Marc is represented as saying:—

"If I kill myself, my suicide is the result, or rather the resultant, of God's will and of mine. In reality, God thinks in us, since our soul is a direct emanation of his essence. If, then, the thought occurs to me to accelerate the moment that I shall quit my actual form, it is to God that I owe it. I have the power, myself, with my freewill to discuss, reject, or admit it. It is with this as with a disease which is insignificant, dangerous, or mortal, and of which the germ is in us. If this thought arises in me without disturbing me, it is insignificant; if it inspires me with a deadly resolution, it is dangerous; if it seizes upon me to the extent of forcing me to carry out my resolution, it is mortal."

The topics of this conversation amid the burning sands of the desert, and the locality in which it is held, notwithstanding the accessories of chibouques and coffee, inevitably recal (to the English reader at least) that discourse which Milton represents as being held upon the fiery floor of hell, and in which were discussed—

> "——— Providence, fore-knowledge, will, and fate;
> Fix'd fate, freewill, fore-knowledge absolute."

It is no part of our object to discuss the different sentiments and opinions expressed by Jean Marc. We seek simply to describe these sentiments and opinions; but apropos of the notion that the soul is a direct emanation of the Deity and the consequence derived from it, we may fittingly refer to an observation of St. Augustine. He has happily characterized as a "strange madness" that state of mind in which, according to the doctrines of the Manichæans, he at one time held that he was that by nature which God was, imputing to the Deity the liability to err, rather than confess that he had gone astray voluntarily.*

A day's gossip, and each traveller takes his separate way. After long travel, our author, returning to Paris, and not forgetful of his "improvised companion" in the desert, seeks for him. He hears of him from many persons, and in no flattering terms; but cannot meet with him. One speaks of him as a madman, another as a bear, a third as an original, but a fourth most aptly describes him as a "useless being, a natural son of René, reared by Antony and Chatterton." Presently, however, our author receives a letter from Jean Marc himself, written on the eve of suicide, and enclosing a will by which he appoints him his literary executor. The letter contains the moral of the book:—

"Is it fatality? is it freewill which urges me?" [writes the suicide] "I know not. What is certain is that I am weary, and that I will have done with it. When you receive this letter, all my doubts will have been cleared up, and I shall, perhaps, at last have comprehended the End and the Cause. You will probably remember our conversations in the desert. Is suicide permitted, or is it prohibited? Am I right, or am I wrong? The fact is that I am about to kill myself, that is all.

"If you have the courage to read the notes I send you, you will learn how it happens that although barely thirty I have come to this. . . . . Amidst their confusion you will discover this terrible truth, the foolish negation of which brings about my death this day,—that under penalty of unhappiness, it is requisite to follow the exemplary precept which God has laid down in Genesis: 'Sous peine de mort, il faut travailler.'

"I have imprudently consumed in one hour, by a useless brilliancy,

---

* Conf. B. iv. sec. 15.

the oil of the lamp which should have burned throughout the night. The darkness has come, and I fear the phantoms. Thus like an infant I throw myself boldly into the arms of my nurse that she may appease my terrors. But I have interrogated in vain the silence and the obscurity, and I can discover no one who can succour me. I depart for future creations, where doubtless I shall live with the experience gained at the price of many miseries. As I have said, I have laid hold of existence on the wrong side, and hence it is that I die disgusted with life without having ever lived. God pardon me, since I was ignorant. . . . . Do not pity me; I die with indifference if not with joy, and I feel a great buoyancy of spirit. Few will miss me, and they will hasten to forget me, so that they may quickly relieve themselves of the little portion of pain I may have left to them. I cannot demand a single tear; I know this, and I am pained with it, because I do not escape from that imperishable egotism of the human heart which longs to survive, a loved being, in the regrets of others—regret, that egotism of the living abandoned by the dead. . . . .

"Before I finish listen to the counsel of one dying: Work; work without ceasing, work without relaxation, with or without result matters little: but, work! Work is the club of Hercules which crushes every monster."

This is the moral of the book; truly, a noble one, if rightly understood, and looking well, tricked up with a scriptural text. But mark the sequence:—

"And now," he writes, "that I am about to receive upon my lips the cold kiss of death, now that all is ended and that in one hour more I shall be stretched out a bloody corpse, if it is asked what thought, what regret, what aspiration opens its wings in my heart? I answer: How happy must that man be who is caressed by a young and lovely wife, and who hears an infant lisp 'Father!' He dwells in the country; a verdant sward stretches before his house; when he walks out his wife is at his side and a great dog follows carrying the little child: he knows the joys of family life. How happy must he be who watches through the night, bent over a book, his head full of thought! Ever and anon he rises to gaze upon strange mixtures fermenting in crystal vases: he knows the joys of a scientific life. Happy is the painter working palette in hand! the statuary who chips the marble! the composer who grows pale listening to the melodies which are sung in his soul! the writer who dresses his thoughts in magnificent forms: they know the joys of an artistic life. Happy is the captain whose splendid costume attracts the eyes of the women! He commands soldiers as brave and obedient as himself; he would die bravely to save the frontier or to prevent a dog entering the Tuileries: he knows the joys of glory and subjection. Happy the Secretary of State who unseals despatches that he does not understand, and signs papers that he has not read! He kisses his hand graciously to the ladies; he speaks not, in order that he may have the aspect of reflection, and bends lowly before the powers that be, because he wishes to become minister: he knows the joys of ambition. Happy the banker who jots down his figures, counts his money, lovingly regards the fifty locks of his strong box, and gains sixty-five per cent. in the most honest fashion possible! He knows the joys of riches. Happy the young man who, when the night comes, with beating heart and light foot, heedless of difficulties, hastens to her he loves and by whom he is loved! He knows the joys of love. Happy all those who are not as I am, all those who are not gnawed by the devouring inquietudes of hopeless dreams."

From this confession we are to learn (and the author's explanation is certainly needed) that the suicide has at length comprehended the impiety of his life, although not of the act by which he is about to terminate it. But how strange the conceptions, how wretched the caricature, of the duties and requirements of manhood! It would be most natural to conceive that the picture here presented to us is a feeble satire; but, as we shall see more fully in the further analysis of this strange work, the artist is drawing in good faith: and the vapid sentimentality is peculiar to the school of literature to which our author's work belongs; while the irony is a characteristic of his hero, who is represented "as adding a corrective, often bitter, to whatever admiration he expresses."

In the foregoing fashion, Maxime du Camp has depicted the matured opinions of his hero. In the subsequent portions of his work the hero is represented as himself recounting, in a series of desultory letters or recitals, the genesis of these opinions, and the history of his life. We shall take up that history at the point where he is left an orphan.

He is still very young, and while suffering from the acute grief consequent upon the loss of an over-fond mother, he is consigned to one of those vast schools where the individual child is left to accommodate itself as best it can to stern general rules and harsh task-work. Naturally delicate and sensitive, crushed by the loss of his home and by the want of all kindness, he becomes moody and retiring, avoids the rough sports and resents the thoughtless indifference of his comrades. He cherishes his griefs apart, and his soul revolts against the unreasoning punishment which follows every shortcoming, however trifling. Presently his feelings assume a more active character, and a bitter hate is engendered in his breast against his masters. He becomes obstinate, heedless, and disorderly, and in the end is looked upon as a hopeless subject, and is opprobriously designated by his teachers, a *cancre*.

But few pleasures brighten his early school days, and these are solely of the imagination. A young comrade, a Corsican, and a somewhat similar spirit, would often while away the hours of rest with wild stories of brigands and descriptions of his sunny home on the shores of the Mediterranean, and thus excite in his listener's breast immoderate longings to escape from the torturing confinement of school. As he grows older, he presently learns the irresistible charms of romance, dramatic and poetic literature; and, aided by his pocket-money and a readily-acquired trick of smuggling the prohibited books into the school, he indulges without stint in the delicious fictions of the poet and the novelist, and creates for himself an ideal world, which more and more disgusts and renders him discontented with the real.

Even in the lad we thus distinguish some peculiar characteristics of the man. The picture is a sad one, but it must be confessed a true one, and one full of instruction.

Time passes on, and he is in his sixteenth year. A riot, brought about by the senseless severity of one of the masters, breaks out among the scholars, and the ringleaders, of which our hero is one, are severely punished. They determine to escape, and Jean Marc and two others succeed, and for a day and part of a night they are afloat in Paris, subsisting as best they may on the scanty money which they have in their pockets. At midnight they rest themselves on the parapet of the Pont Neuf, wearied and dissatisfied with a day of freedom, in which they had not known what to do with themselves, momentarily dreading capture, and not knowing how to act on the morrow. They sit there long, depressed and silent, each occupied with his own fears, when Jean Marc, glancing at the swift stream beneath them, exclaims, "Why not *die?*"

It was under these circumstances that the first thought of suicide entered into his mind; from this time often to recur. Here it was the passing thought of a frightened lad, whose head was teeming with the wild notions of romance. But when next the idea occurs, it assumes altogether a different and more determinate aspect.

Before daybreak the boys are captured, and each imprisoned in a separate cell, but not before Jean Marc, irritated by a remark, had struck the master of the school over the face. This, of course, places him beyond the bounds of mercy, and for ten days he is kept in solitary confinement. With a refinement of cruelty he is suffered to appeal again and again by letter to an aged aunt, beseeching her to remove him from the school, but these letters are never forwarded to their destination. Ill from his confinement, and despairing at this seeming utter desertion, he gives himself up to thoughts of suicide, and contrives to secure the means for effecting the act, if driven to extremity. At length his aunt appears upon the scene, and he is transferred to another college, where he finds masters who at least have the merit of being polished, where every care is bestowed upon the pupils, and where there is a species of family tuition. But the mental mischief which had been done was too deep to be eradicated, although its effects were somewhat modified by the happiness of his present lot.

"It was while in confinement," he writes, "at war with the brutalities of a stupid and ignorant master, attended by domestics that a recruiting sergeant would not have enlisted, and who ridiculed me, that I first understood that my nature was too haughty, sensitive, and unsociable, in consequence of an exaggerated delicacy, and was not fitted to shoulder humanity with impunity. I utterly disdained all who surrounded me. Society seemed to have united its forces to crush an infant, and, as Jean Jacques, I was ready to cry—*Carnifex! carnifex!* I hated equally every one in the school, which for me was the universe. I hated the master who kept me in this absurd prison; the attendant who had charge of me; and my comrades, who had not attempted to release me, but had cried—*Væ victis!* Be it so: I will heed them no more; I detest the former; I scorn the latter. I will go on my way alone, pressing forwards in loneliness and pride. During the ten days I was confined, my brain received the germs of many thoughts, which have since been matured, branching throughout the mind like thorny brambles. I imbibed then an aversion to brute force which has never quitted me; and I abominate the useless power of athletæ, of rich men, and soldiers."

He is now seventeen, and he yields himself up to vague dreams of glory, of pleasure, love, and poetry. He idolizes *Antony* "who has spitten in the face of society," and *René*, the sceptical Christian, the visionary, and the poet, and already he had learned from the pages of Gibbon to admire Mahomet. In this fashion he panders to his worse traits. Presently he becomes his own master, and at once plunges into the vortex of a so-called life of pleasure.

"I was attracted towards everything by an immoderate curiosity: I sought to learn, and I learnt at my own cost. I lived prodigally and without bounds, holding back at nothing. I was at length free, and realized the dream of my life, and like those who, after a long fast, gorge themselves imprudently with food, I devoured, even to death. Horses, hunting, women, nocturnal orgies, the theatre, Paris insensate, follies of every kind, extravagances of every sort, absurdities of every species, and the foolish satisfaction of a stupid self-conceit. One day, at sunrise, I crossed swords with an adversary; I felt the cold iron penetrate my arm and tear my chest, whilst he fell, uttering a great cry. 'Two true men nearly killed for a girl,' sneeringly remarked a bystander.

"Scarcely well, and proud perhaps of carrying my arm in a sling, I recommenced my shameless life. The Bois de Boulogne saw me every day, the coulisses every evening, the Cydalises every night. *I marched rapidly towards the gulf, not of ruin, which is nothing, but of brutishness, which is much worse.* Lastly, ennui came, ennui profound, implacable, infinite. I apprehended, from the disgust which seized me, the foolishness of which I had been guilty. I withdrew quickly from this absurd, mad, and wicked life, in which I had wallowed nearly a year; an existence impious and abominable, *which lays hold of a man, and transforms him into a cretin,** as the sea which swallows a living body and casts up a corpse."

He next flies to a distant mountain village, and seeks there to repair the evil effects, physical and mental, which his excesses have induced. Recalled to Paris, on arriving at his majority, he is bewildered by business matters, his regrets for the past, his cares for the future, his physical sufferings, his indecision upon the choice of a career, and his isolation.

"The study of law, to which it was sought to lead me [he writes], frightened me by its dryness, its prosaic nature, and frigidity. My spirit, naturally contemplative, was inclined towards artistic matters; but, from a sentiment of false dignity, I refused to fix myself to any course assiduously, and I obstinately and angrily persisted in doing nothing. I lived in an idleness one hundred times more dangerous than the most dangerous work.

"I was too much ashamed of my first outset in life ever to fall back again into the bottomless abyss of debauchery and folly. Some occupation was, however, needful for me, and unhappily I found it in myself. It was determined by my pitiful organization. It was the consequence, perhaps inevitable, of those tedious maladies which had assailed my childhood, and of the sufferings which they had bequeathed me. I became—the word is so pretentious I hesitate to write it—a dreamer. Daily stretched on a couch, motionless and with drooping hands, the eye lost in strange contemplations, I fell into boundless reveries, on being aroused from which all reality pained me. I lapsed into another and more attractive life, attaching my thoughts to everything which occurred, and converting all into aliment for the insatiable demon which inhabited me. Pleasant days passed away thus.

"Sometimes I bordered upon ecstasy, but sometimes also I suffered considerably. When my spirit, which, as holy men say, could not see the things which were beautiful, followed the ways of sadness that its perilous inertia had opened to it, I suffered intolerable pains. Unceasingly enticed by these singular attractions towards the chagrin which disturbs enfeebled and nervous natures, I loved the ill which devoured me; I sought for and provoked it, abandoning myself to it without measure; I experienced the invincible attraction of suffering; my pride was gratified by it, and I hunted my soul violently in the sombre depths of imaginary torments.

"My desires, even the most legitimate, fell into an ever-agitated river, and were cast up again upon its banks dead or dying; and I relished these dangerous joys, not doubting that I was yielding up my being to a pitiless vampire.

"Naturally reserving to myself the best part among the personages with which I peopled this ideal life that I had created, I quickly

* The Italics in this quotation are our own.

became disgusted with the banal outer life, which clashed with my instincts, or, at least, with my susceptibilities. I separated myself then from all society, and lived nearly alone, seeing but a few of my friends who were indulgent towards me.

"I felt presently the danger of this passion of mine, a hundred times more formidable than drunkenness, because it is a perpetual intoxication. I had developed certain of my intellectual faculties, but I had neglected others, and I had cultivated my sensibility to so great a pitch that everything irritated and pained me. I sought to end this malady at a single blow, before it became mortal, and so I resolved to take a long voyage."

Starting from home, he seeks distraction in the East, but in vain. He had departed unsociable, he returned more so; he had gone away suffering, he returned incurable. He now seeks refuge in suicide by poison; but too eager to attain his end, he overdoses himself, the stomach rejects the deadly agent, and he escapes.

What follows of our hero's life are chiefly incidents of pellicacy* and adultery, decked up with a wealth of meretricious and *quasi*-virtuous sentiment. With these details we have nothing to do, further than barely mentioning them. We pass on to show the character of the pseudo-philosophy which pervades the book, and by which all distinction between virtue and vice is done away with, and the former term rendered an unmeaning one.

Jean Marc, it must here be remarked, does not so much philosophize himself as adopt the philosophy of others to practical and every-day uses. He has a friend named Sylvius, to whom he is accustomed to defer in matters of recondite thought. This friend is mortally wounded in a duel, arising out of a squabble in the lobby of the Opera, and on his death-bed he takes occasion to recount his Belief, and in so doing sums up the philosophy of the book. The whole scene in which this occurs is singularly curious as a piece of contemporary writing, as a psychological study, and as an expression of opinions, which seem largely to pervade much of the popular French literature current in England. We shall describe it, therefore, somewhat fully. At a time when fears have been aroused that not merely the doctrines of the Church of England, but the primary tenets of Christianity are being tampered with, by certain publications foreshadowing a school of extreme idealogical theology, it cannot be useless, apart from any other considerations, to note what an extravagant and fantastic ideology has wrought among a highly cultivated class intellectually (a small class it may be, but an influential one, nevertheless) across the Straits. In their concentrated form, the doctrines put into the mouth of Sylvius might seem to the English reader to partake of the character of an absurd, although clever caricature. But when it is considered how aptly these doctrines when diluted with sentiment, and spiced with garbled Scripture texts, appeal to the passions without unduly exciting the prejudices of the young, it will be conceded, we think, that their absurdity is no more an obstacle to their being largely received, than the absurdity of the most glaring system of quackery is an obstacle to its being largely indulged.

Sylvius is wounded fatally. A priest (summoned by an over-officious servant), the doctor, Jean Marc, and Bekir Aga, his Arab servant, stand near the bedside of the dying man. "My son," said the priest, "doest thou wish to be alone with me that thou mayest confess thy sins, in the name of the Father, the Son, and the Holy Ghost?" "No," replied Sylvius; "what I have got to say all the world may hear." "Believest thou in the Catholic, Apostolic, and Roman religion?" rejoined the priest. "It was the religion in which I received baptism, and in which I was brought up," answered Sylvius; and then, rejecting the prayers of the Church, he exclaimed, "I believe in God, the Father and Creator Omnipotent; I believe in the life eternal; but I do not believe Jesus Christ to be the *only* Son of God." "My child," cried the astonished priest, "believest thou not in the divinity of our Lord Jesus Christ?" "I believe," replied Sylvius, "in Jesus Christ as an Apostle, Prophet, and Word of God, but not as the only Son of God." Whereupon Bekir Aga who had been listening attentively to this conversation, interposes, crying out, "Thou art right! thou art right, friend of my master! Has not the prophet (Mahomet to wit) said, 'Blaspheme not God in saying that he has children.'" And then, after reciting sundry dogmas of his faith, Bekir Aga briefly describes the sensual delights of the paradise promised to all true believers, and adds: "God is generous, His mercy is infinite, and thou mayest enjoy all the felicity that He has promised to the sons of Islam, if thou wilt say with me, from the bottom of thy heart: 'There is no god but God, and Mahomet is His Prophet!'"

The priest drew back indignantly, but Sylvius, smiling, said: "O Bekir Aga! I can say with thee 'There is no god but God, and Mahomet is His Prophet;' but I add that there have been and will be many other prophets of God." "What! do you admit the fatalist doctrines of this miscreant?" passionately exclaimed the priest. "Does not your reason revolt against the lying fatalism which is the foundation of Islamism?" Bekir Aga, quickly interfering, responded, "Deniest thou the omnipotence of God, in believing that any fact, however insignificant it may be, can occur without his express permission?"

Sylvius, turning towards Jean Marc, here playfully remarked, "Seest thou not that I resemble Robert the Devil, when, in the fifth act, he is pulled about by Alice and Bertram." Then raising himself painfully the dying duellist speaks, and the scene proceeds as follows :—

"'I believe [said Sylvius] in my freewill, force interior and personal, which enables me to direct my thoughts and my actions; I believe in fatality, force exterior and foreign, which weighs upon my thoughts and my actions, dragging them out of the path that I have freely chosen: I believe in the necessary and indispensable existence of the one and the other, because, between the two, at a variable distance, is stretched a line, that of Providence, which it is necessary to follow, and which comes naturally to us. This is a mechanical theorem which can be demonstrated by the parallelogram of forces. If I obey solely the *objective* (fatality), or solely the *subjective* (freewill), I withdraw from the truth, the wisdom, the virtue, the reward, which form the immovable, intermediate line in the Lord's will. My freewill can constrain and vanquish fatality; it can, in certain moments of passion and of ecstasy, constrain even God to draw it to himself. I believe, then, in freewill, in fatality, and in Providence.'*

"The priest and Bekir Aga looked at each other with eyes of which the astonished expression proved that they had not comprehended the meaning of the words uttered by the dying man. Nevertheless the priest, desirous to discover the yielding side of this spirit so rebellious to his efforts, said with emphasis: 'thou, at least, believest in the immortality of the soul?' 'No,' answered Sylvius, 'I believe in its eternity!' 'This unhappy man,' said the poor priest, turning towards us, 'has been poisoned by the subversive doctrines which have already done so much evil in our epoch.' 'It is permitted to all to seek the truth,' I responded. 'You are impious,' he murmured.

"A strange animation brightened the visage of Sylvius: he moved his lips as if he recited some prayer. All were so silent that the ticking of thetimepiece was heard. 'Raise me,' he said; 'I would again speak.' We surrounded him, tossed up the flattened pillows, and the doctor and myself supported him upon our crossed arms. He re-

---

* It may be said of M. Sylvius as of Hudibras—

> "He could reduce all things to acts,
> And knew their nature by abstracts;

And in the sequel it will be seen that he knew—

> Where entity and quiddity,
> The ghosts of defunct bodies fly;
> Where Truth in person does appear,
> Like words congeal'd in northern air.

Like Ralpho, M. Sylvius is also—

> "Deep-sighted in intelligences,
> Ideas, atoms, influences;
> And much of terra incognita,
> Th' intelligible world would say."

Abstract metaphysical terms, such as *subjective* and *objective, Ego* and *non-Ego, monad,* and so forth, become under his manipulation distinct entities.

mained a few moments immovable, as if absorbed in thought, and then said:—

"'No! I am not impious, because I believe in thee, O my God! Source of all virtue, of all truth, of all intelligence, of all justice, and of all mercy; I believe in thee! Thou art in us as we are in thee, all are in thee as thou art in us. O compassionate Father! thou art the life eternal which circulates in all creation, and even in those subtle perfumes which are perhaps odoriferous animalcules. It is thy mediation among the things of nature which makes them so beautiful; it is thee, always thee, that we search for, that we love in the landscape, woman, the stars and the blue sky; it is that we may incline to thee, that we may draw nigh to thee, that we may better comprehend the mysteries of thy infinite space, that increasingly we strive to augment our intelligence and our mind. O, my God! I believe in thee. Thou art the Idea,—power indestructible, invincible, persistent, unchangeable, always increasing, expanding, and strengthening, mother of faith, of hope, of charity, and of restoration, agent mysterious who speaks in the conscience of each, and who embraces the heart of all; fluid impalpable, which nothing can render motionless, which advances slowly but inexorably towards its end, and which draws everything to its aid, even its enemies—obstacles and persecutions; thou art the Idea,—fertilizing river which streams into humanity, and which penetrates it as water penetrates a sponge! Thou art Love,—attraction irresistible, which makes tremulous the molecules of thy essence diffused throughout the great whole, and which successively draws one towards another, in order that the two portions of thy power may reunite momentarily in a union full of ecstasy. This ecstasy materialists have termed intoxication of the senses, and it is, perhaps, the beatitude of thy vibration revealed in us! O, my God! I believe in thee.

"I believe in thee, who knows all things by thy sovereign memory and sovereign prescience; I believe in thee, author of progress, in thee who brings forth the best effects from the worst causes; I believe in thee, who with thy divine finger points out to us the future, and who destroys not the past that it may serve to ameliorate the future, because thou art the Law, Father of justice! and thy power is never retroactive; I believe in thee, thou art the soul in which we live, thou art the soul who lives in us; I believe in thee, I believe in thee!

"I believe in my soul,—emanation essential of God, integrant part of him, and divine as he is divine; I believe in my soul, immaterial and progressive in its nature, intelligent in its operations, eternal in its destiny!

"I believe in my soul,—endowed with ubiquity, since it exists easily in many places at one time; in the hearts of my friends, in the soul of my mistress, in the remembrance of those who are afar off, in the animals which serve me, in the landscapes I love, in the oceans I have traversed, in the stars that I look upon, in the deserts where I have slept, in the dead who have gone before me!

"I believe in my soul,—aggregation of divers monads, legion composed of different essences derived from the other souls that I have met with, loved or hated, vanquished or aided, lost or saved in my previous existence! These portions of the soul are each in themselves as a soul; these are the depositaries of the reminiscences of my former lives, and are the source of my anticipations, my sympathies, and my innate ideas; these, by turns, according as they are stirred up, look out by my eyes, and give them their variable expressions of wickedness, softness, anger, charity, courage, fear, goodness, and tenderness. They are united in one as a sort of deliberating assembly, which discusses, judges, directs, condemns, approves, corrects, restrains, excites, excuses my thoughts and my actions. Each gives its reasons for or against, and the result is determined by the plurality of voices, except when an unforeseen and grave circumstance suddenly gives rise to an unanimous decision, induced by the irresistible eloquence of one of the interested monads. Then, as good men say, I yield to my first impulse. It is this aggregate which, always increasing in intelligence and number, constitutes my eternal soul.

"My soul is eternal; it always has existed and always will exist.

"It has lived already under a palpable form, and it will live again; it will advance, climbing the ascensional scale of intellectual aggrandizement; when it becomes the most elevated monad of this planet, it will foresee the near coming of new times, it will hasten the march of humanity illuminated by its rays, and will carry it away in its train towards the superior monads, to which we shall journey all together, to enjoy more perfect and more numerous senses, a greater multiplicity of and more vivid sensations, a loftier reason, and more extended comprehension; it will guide its sister monads, divested of their prevaricating instincts, towards the essence even of God, who is justice, intelligence, truth, and love supreme.

"Happiness in this life is an insignificant thing with God; intelligence alone, and the virtues which arise from it, are of any worth in His eyes. The more intelligent a man is, the nearer he is to the Lord, the nearer to Beatitude.* What signifies unhappiness and misery? Are they not the fires which purify the metal? Intelligence, the gift direct of God, is the recompence of work accomplished in preceding existences; it alone agrees in following the Providential line; other guerdons are often on the route of freewill and fatality; happy he who has both the one and the other in his portion. It is said of poets and apostles that they are above humanity: this is true. The divine path in which they pacifically advance predominates greatly above all the mortal interests of the *Ego* and *non-Ego*.

"I believe in the persistence of the *Ego*,—force latent of which I am assured, and which sometimes surges up in all its clearness; conscience inactive, but always living, and which is aroused on the day when

---

* *Parson.*—"Intellectual power, refined to the utmost, and wholly void of beneficence, resembles only one being, and that, sir, is the Principle of Evil."

*Randal* (startled).—"Do you mean the Devil?"

*Parson.*—"Yes, sir—the Devil; and even he, sir, did not succeed! Even he, sir, is what your great men would call a most decided failure."—*My Novel*, b. ix. c. viii.

death renders itself mistress of the body. I shall die; I am about to die; that is to say, I am about to undergo a new transformation. Then my soul, divested of this fleshy covering which imprisons it, and which it now wishes to cast aside, my soul once more enters fully into the exercise of its *Ego*, comprehending fully all the progress that it has already made, perceiving that which it has still to make, rendering to itself account of effects and causes, and imprisoning itself joyously in another body, in order to continue the work for which God has chosen it.*

"I believe in the Providential mission of self-denying men, apostles, and prophets, who have raised the human spirit by teaching higher morals, and who have scattered among their fellow-mortals seeds, the fruits of which generations yet to come will harvest. I believe in these men,—in Zoroaster, Menu, Abraham, Moses, Confucius, Socrates, Jesus Christ, Manes, Mahomet, Luther, and many others. I believe in those whom I have seen in our own days, gentle, benevolent, peace-makers, reinstating the flesh and fertilizing the spirit, and who have been loaded with outrages and become martyrs as the Son of Man. I disbelieve entirely those senseless bugbears of eternal pains, of flaming hells, of horned devils, and of Satans cursed for ever, a laughable phantasmagoria made use of by the wicked to terrify the people. I believe in a God of indulgence and of mercy; the God of vengeance is dead and will revive no more; the times of wrathful and terrible divinities are gone by; unpitying heavens have passed away for ever; Jehovah Sabaoth is now without hosts, and the blood of his Son no longer suffices to quench the thirst of panting humanity.

"I would say the *prayer* which Jesus taught his disciples on the dusty roads of Palestine—the prayer of those who love, of those who believe, of those who suffer, of those who hope."

And so, repeating the Lord's Prayer, Sylvius ends his confession of faith.

The author of the essay in the *Connoisseur* (No. 74), on the Modesty of the Moderns, in including all the Vices instead of Virtues in the character of a fine gentleman, suggested the writing of a new treatise on Ethics, or a System of Immoral Philosophy. It would seem, judging from the creed of the dying duellist, as if M. Maxime du Camp aimed at being the promulgator of such a system. In an early number of the *Connoisseur*, moreover (No. 9), in an essay upon the impetus given to "free-thinking" by the publication of Lord Bolingbroke's works, is to be found, also in the form of a creed, a satirical summary of the tenets of the religious sceptics of that day. In this creed the free-thinker is

* Cyrano-Bergerac, in his vision of Hell, states that he found Pythagoras, much to his discontent, associated with the comedians. It is marvellous to conceive how modern advocates of the doctrines of metempsychosis can overlook the essential nature of this connexion. See the *World*, No. 163; the *Doctor*, chap. 127; and Dr. Pusey's edition of *St. Augustine's Confessions*, Appendix, Note iii. a.

represented as avowing his belief in the first philosophy, but not in Moses; in Mandeville, Hobbes, Shaftesbury, and others of the same category, but not in the Evangelists; in Bolingbroke, but not St. Paul; in tradition, but not Revelation; in the Talmud and Koran, but not the Bible; in Socrates, Confucius, Sanconiathon, and Mahomet, but not in Christ. Finally, he asserts his belief in all unbelief.

A comparison of this creed with that of the dying duellist (fitting apostle of the faith he professes!) brings into clear light the special peculiarity of the Werther school of moral philosophers. While adopting all the essential tenets of the former creed, with a refined, Mephistophelian ingenuity, it dresses them in a quasi-Christian phraseology, and includes among them certain theistical notions. The result is certainly a most incongruous one, and the idea of Deity a complete negation. In truth, the pseudo-philosophical doctrines of Sylvius constitute an "eclectico-pantheistical farrago," which, in reality, is the rankest atheism, clad in an ill-fitting guise of spurious Christian æstheticism.* "The whole mind of the world," says Cousin, "is not able to vindicate pantheism. One attempts it in vain. If he be consistent, he ends with it only at a species of soul of the world, as the principle of things; at fatality as the only law, at the confusion of good and evil—that is, at their destruction, in the bosom of a vague and abstract unity, without a fixed subject."† "What is pantheism?" writes Pascal. "It is not an atheism disguised, as they say: no; it is an avowed atheism. To say, in presence of this universe, so vast, so beautiful, so magnificent: God is there entire, behold God, there is no other; is to say as clearly as possible that there is no God, for it is to say that the universe has not a cause essentially different from its effects. . . . However immense it may be, this world is finite in itself, compared to God who is infinite; he manifests, but he also veils his grandeur, intelligence, and wisdom. The universe is the image of God, it is not God; something of the cause passes into the effect, it does not exhaust itself there, and it remains entire. The universe is so far from exhausting God, that many of the attributes of God are there covered with an obscurity almost impenetrable, and are discovered only in the soul of man. The universe is a necessity; but the soul is free, it is one, simple, essentially identical with itself, under the harmonious diversity of its faculties: it is capable

---

* "Who," asks St. Augustine, after attacking the Manichæan theory—"who would not execrate these things? Who would not understand them to be injurious and abominable? But they, when they catch men, do not utter these things at first, for so should they be laughed at, or avoided by all; but they choose passages out of Scripture, which simple men do not understand, and thereby deceive unskilful souls."—*De Agon. Christian.* c. 4.

† *Cours de la Philos. Mod.*, Lect. V. p. 3.

of conceiving virtue, and of accomplishing it; it is capable of love and of sacrifice. Now, we are averse to believing that the being who is the first and last cause of the soul is an abstract being, possessing less than he has given, and having neither personality, nor liberty, nor intelligence, nor justice, nor love. Either God is inferior to man, or he possesses at least all that is permanent and substantial in man, with infinity besides."*

In what manner the pseudo-philosophical doctrines, of which Sylvius is the mouthpiece, and Jean Marc the devoted disciple, are adapted to every-day uses, we have now to learn. This portion of our task must, from its nature, be effected with brevity. And, first, of the mode in which the problem of happiness is solved.

Sylvius, in a letter to Jean Marc, tells us that every one has an indefinite sentiment of inquietude which urges him at all times to seek novelty. This sentiment he calls the "right to happiness." We all possess this right, we know it instinctively, and, consequently, we seek to be happy. If we seek for happiness in ourselves, we shall not find it; if we seek for it in others, we shall be also unsuccessful. It is to be found only in what he calls the "line of Providence," which lies equidistant (as explained in his creed) from the *ego* and *non-ego*. In fact, it is to be found in Love, which is an exchange between our own forces and those of others. Equal communion of the *objective* and *subjective* is requisite, we are told, to make us happy. In the search after happiness, however, we often deceive ourselves; hence it is that women become mobile, men unfaithful. From this point of view, we are not surprised to learn that the legendary history of Don Juan is a symbol—that it is, in fact, a sacred tradition, of his school of morals. "He marched on," writes Sylvius, "from amour to amour to the conquest of an ideal, which he foresaw. Many brave men, of the number of those to whom Rabelais has dedicated his works, have seen in this ideal solely a coveted mistress. He sought happiness where, in reality, he ought to have manifested it, in love; he did not find it, and he again went in quest of it. Rest assured, that he has since found it in the world he now inhabits. I believe that in the rewards of the future, love will be recompensed as a virtue. God is a part of ourselves, and he is not forgetful, believe me, of the happiness that we have given in rendering ourselves happy. True felicity lies in this path. The Master has said, "Love ye one another." But it has been said in the creed, that happiness in this life is insignificant with God compared with intelligence. How, then, consistently (not that consistence is at all needful) represent God as mindful of

* *Des Pensées de Pascal*, avant-propos.

our happiness in love, and love as happiness ? Love, we must now learn, is the mainspring of intelligence. It is the "armour and armature of man."

"March onwards," writes Sylvius in the same letter, after remonstrating with Jean Marc for feeling grief and an increased hopelessness at the loss of a mistress, and instructing him that it was only by successive and repeated trials that he could hope to attain to a true knowledge of love—"march onwards towards the new hope, towards the increase of all thy faculties—that is to say, towards love. Learn how to love, and give thyself to it without measure ; thy heart will beat more easily, thy breast expand more fully, and thy brightened intelligence will blaze upon the forehead like a star."

We need not do more than note briefly without polluting our pages with details, how by a logical sequence pellicacy becomes the chief social development, the "mistress" the high priestess of this *cultus:* how prostitution has a scintillation of divinity about it (—"The Saint-Simonians* are right," exclaims Jean Marc, apropos of this subject, "the name of God is written upon every wound.... Let us love, and leave the rest to God."—), and how even adultery must be added to the list of virtues :—"Above the narrow and abusive right of a husband (it is Jean Marc who again speaks,) there is a right, human, emotional, imprescriptible ; and this right I use, without scruple, with the profound conviction of remaining an honest man."

It is an unpleasant reflection that the two most popular works in the French tongue inculcating these doctrines, *The Lady with the Camelias,* by A. Dumas the younger, and *Fanny,* by L. Feydeau, brilliantly written stories, have been recently translated into English, and are now widely circulated in this country. Upon the first work was founded the opera of *La Traviata,* and the story (which is simply the apotheosis of a common prostitute) is pretty accurately epitomized in the libretto. The production of *La Traviata* in London, and the avidity with which it was (and is, indeed, still) received on all hands, gave birth to a widespread desire for, and led to the publication of, the original story; and this, again, has led to the translation of the even still more detestable and abominable work of M. Feydeau. Truly Mephistopheles must have once more occupied the prompter's box.

Divested of all periphrasis, the character of Jean Marc is such as St. Augustine so exquisitely described his own character to

---

* "Pantheism is, properly, the deification of everything, the great whole regarded as God, the universe-God of the most part of my adversaries, of St. Simon, for example. It is at the bottom a veritable atheism, with which one can mix, as St. Simon has done, at least his school, a certain religious colouring."—*Cousin, Op. cit.* Lect. V. n. 3.

have been when young. "Out of the muddy concupiscence of the flesh," he writes in his *Confessions*, "and the bubblings of youth, mists fumed up which beclouded and overcast my heart, that I could not discern the clear brightness of love from the fog of lustfulness. Both did confusedly boil in me, and hurried my unstayed youth over the precipice of unholy desires, and sunk me in a gulf of flagitiousness. Thy wrath had gathered over me, and I knew it not. I was grown deaf by the clanking of the chain of my mortality, the punishment of the pride of my soul, and I strayed further from Thee, and Thou lettest me alone, and I was tossed about, and wasted, and dissipated, and I boiled over in my fornications, and Thou heldest Thy peace, O Thou my tardy joy! Thou then heldest Thy peace, and I wandered further and further from Thee, into *more and more fruitless seed-plots of sorrows, and a proud dejectedness and a restless weariness*."*

But with none to attemper his disorder and turn to account these, the extreme points of God's creation,—none to "put a bound to their pleasurableness, that so the tides of youth might have cast themselves upon the marriage shore, if they could not be calmed and kept within the object of a family as Thy law prescribes;" and condemned to that penal blindness which God dispenses to lawless desires,† Jean Marc (as Werther) passes from dejectedness to despair, from weariness to disgust of life. And so to suicide, the natural consequence of a life that in its maturity had attained no higher conception of happiness than mere habit, of the duties of manhood than a barren sentimentalism, of Deity than an utter negation, of virtue than an expedient vice, of law than a conventional virtue.

"The suicide," Aristotle wrote, "does not undergo death because it is honourable, but in order to avoid evil;"‡ and Plato has represented Socrates as saying, addressing Simmias, "Do you know that all others (except philosophers) consider death among the great evils?" "They do, indeed," Simmias replied. "Then do the brave amongst them endure death when they do endure it through dread of greater evils?" "It is so. All, therefore, except philosophers, are brave through being afraid and fear; though it is absurd that any should be brave through fear and cowardice."§ The suicide, in fact, believes death to be an evil of less magnitude than the ill from which he seeks to escape. This

---

* *Confessions*, Bk. ii. c. 2.

† "How deep are Thy ways, O God, Thou only great, that sittest silent on high, and by *an universal law dispensing penal blindness to lawless desires*."—*Confessions*, b. i. c. 18.

‡ *Ethics*, b. iii. c. 7.

is the great principle which governs the commission of self-murder. No influence, therefore, can be conceived more powerful to hold in check the self-infliction of death, than such as indelibly fixes upon the act the character of an evil far surpassing in gravity any, even the sharpest or bitterest ill, to which man may be subjected. Hence the Christian Church, in branding suicide as an inexpiable offence against God, and Christian Legislatures in attaching to it the stigma of an irreparable and disgraceful crime towards man, stamp the act as an evil, morally and socially, to which all others which might prove influential in promoting self-murder are, in comparison, petty and insignificant.

But the first of these motives is entirely, the latter comparatively powerless in restraining disciples of the Werther school of philosophy, of which Jean Marc is the latest example. For, when the idea of Deity is a negation, that of sin also is a negation; and when the conception of crime is not vitalized by that of sin, crime becomes a mere question of expediency. If, then, suicide be regarded solely in this light, the animal love of life, the physical fear of death, and conventional notions of disgrace, will alone interpose to check the tendency to self-murder.

Jean Marc is dead by his own act, and for his epitaph is written:—"*Ici git la dépouille d'une Ame Eternele.—O Mort, que j'ai forcée a m'obeir, déjà je t'ai vue souvent dans mes existences antérieures, et souvent je te reverrai dans mes existences futures. Choisis-moi de préférence lorsque tu voudras déliverer un Homme de l'enveloppe qui embarrasse son ame éternelle; fais que je sois ton élu à toujours, et conduis moi de Transmigrations en Transmigrations jusqu'à Dieu, afin que je puisse rentrer à jamais dans son Essence Infinie et Eternelle! Ainsi soit-il!*"

This is the ideal of suicide.

"*Regardez un peu,*" said the landlady; "*Messieurs, il m'a gaté trois matelas, et il me doit quarante quatre francs.*"*

This is the real.

---

### Art. IV.—FEMALE PHYSICIANS.

Why should woman be excluded from the legitimate practice of Physic? why should she be debarred from wielding pill and potion?—that is to say, according to the rules of art, for it is a painful fact that she is not excluded, neither does she exclude

---

* Thackerah's *Paris Sketch-Book:—The Gambler's Death.*

